# Preparation, Reaction Kinetics, and Properties of Polyester Foams Using Water Produced by the Reaction as a Foaming Agent

**DOI:** 10.3390/polym17091266

**Published:** 2025-05-06

**Authors:** Fabian Weitenhagen, Oliver Weichold

**Affiliations:** Institute for Building Materials Research, RWTH Aachen University, Schinkelstraße 3, 52062 Aachen, Germany; weitenhagen@ibac.rwth-aachen.de

**Keywords:** polyester, polycondensation, bio-polymer, foam, kinetics

## Abstract

This study explores sustainable foamed polyester materials derived from natural or bio-based building blocks, including succinic, glutaric, and adipic acids, combined with trimethylolpropane and pentaerythritol. By precisely tuning the ratio of functional groups, the resulting polymers contain minimal free functionalities, leading to lower hygroscopicity and enhanced stability. The reaction is monitored by tracking the mass loss associated with water formation, the primary condensation by-product, which reveals a first-order kinetic behaviour. Infrared spectroscopy indicates that foaming occurs in a narrow time window, while esterification begins earlier and continues afterwards. Thermogravimetric analysis confirms thermal stability up to ~400 °C, with complete decomposition at 500 °C and no residue. Scanning electron microscopy images of test specimens with varying densities reveal dense, microporosity-free cell walls in both materials, indicating a homogeneous polymer matrix that contributes to the overall stabilisation of the foam structure. In flammability tests, the foams resist ignition during two 10 s methane flame exposures and, under prolonged flame, burn 40 times more slowly than conventional foams. These results demonstrate a modular system for creating bio-based foams with tunable properties—from soft and elastic to rigid—suitable for diverse applications. The materials offer a sustainable alternative to petrochemical foams while retaining excellent mechanical and thermal properties.

## 1. Introduction

Foams are indispensable in today’s world. They can be found in insulation materials, packaging, seat cushions, and mattresses, and as a universal padding material on construction sites [1,2,3,4]. At a time when energy and climate efficiency are becoming increasingly important, the significance of insulation materials cannot be valued enough. Probably the best-known example is foamed polystyrene, either expanded (EPS) or extruded (XPS). These have thermal conductivities between 0.025 and 0.045 W/(m·K) and, therefore, buildings are frequently insulated very efficiently with XPS [5,6,7]. The main disadvantage of these materials is that they are based on petrochemical feedstock, and the circularity suffers from the fact that XPS is difficult to recover from construction waste [8,9]. Additionally, foamed polystyrene is highly flammable and produces burning droplets during combustion [10,11,12].

Another well-known class of foamed materials is polyurethane, which is prepared by the polyaddition of isocyanates to alcohols. In the presence of moisture, the isocyanates hydrolyse to form CO_2_, which acts as a foaming agent. However, the high reactivity that guarantees a broad range of applications comes at the expense of the high toxicity of isocyanates [13,14]. The release of CO_2_ during the reaction also has a negative impact on the carbon footprint of these materials. In addition, the main source of isocyanates is again petrochemical feedstock [15]. For some time, efforts have been made to improve the environmental impact of polyurethanes by partial substitution with renewable materials, such as epoxidised castor and soybean oils, or bio-based polyols [16,17]. However, the isocyanate components are difficult to substitute, so toxicological, environmental, and resource issues will remain [18,19].

Foams made from 100% renewable resources, which are non-toxic and ideally biodegradable, require a fundamentally different approach. One such approach is foamed starch, which is made from an aqueous starch solution and a blowing agent such as steam, and it is already being used as packaging material or insulation [20,21]. However, its insulating properties are somewhat inferior compared to EPS sheets, and starch sheets are relatively brittle. The foams are also sensitive to water, which limits their use to indoor or dry environments [22]. In addition, the large-scale use of starch would ultimately interfere with the food chain, which should be avoided.

This approach clearly shows that water is an effective and sustainable foaming agent. During polycondensation reactions, water is generated in situ as a by-product. This internally formed water evaporates at higher reaction temperatures, which can be used to trigger the expansion of the thermoset polyester resin and the formation of a stable foam structure. By using bio-based multifunctional polyols and carboxylic acids, a variety of sustainable thermoset polymers with tunable properties can be synthesized [23,24]. Among these, the system of citric acid and glycerol has been extensively investigated [25,26,27]. Due to the trivalent functionality and high reactivity of citric acid, highly crosslinked and mechanically stable polymers can be produced. At elevated temperatures, the water formed during the reaction is released as vapour and acts as a natural foaming agent to produce polyester foams. Adjusting the ratios of acid to hydroxy groups allows control of the mechanical properties. However, this comes with the disadvantage of leaving free functional groups after the reaction, which renders the products hygroscopic [27]. Modifying the materials’ properties while maintaining a 1:1 stoichiometry of functional groups requires an extended set of compounds. At the same time, the different reactivities of primary and secondary carboxy and hydroxy groups in the citric acid/glycerol system can lead to major variations in the product properties, even with minor variations in the reaction conditions.

Based on these considerations, we present a detailed study on a set of polyester foams based on linear dicarboxylic acids and multifunctional primary alcohols. The selection of primary alcohols enables uniform reactivity and a controlled polymerisation process. Concurrently, the systematic variation of different bio-based dicarboxylic acids allows for targeted modification of the polymer backbone, leading to tunable material properties, such as thermal and mechanical stability. The kinetics of network and foam formation, as well as the foam properties, are correlated with molecular properties such as the chain length of the dicarboxylic acids (succinic acid, glutaric acid, adipic acid) and the crosslinking density (trimethylolpropane, pentaerythritol). It was chosen to keep the ratio of functional groups at 1:1 to avoid imponderables in reactivity and an excess of polar functional groups in the final polymers, which could trigger hygroscopic effects. The detailed study of the kinetics allowed for the scale-up and improvement of the manufacturing process. Furthermore, parameters influencing the density, thermal stability, heat conductivity, as well as flammability are studied.

## 2. Materials and Methods

**Figure 1:**
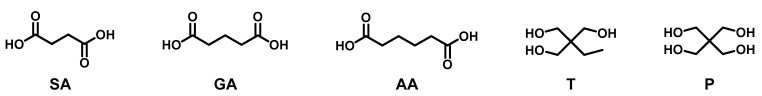
The set of acids and alcohols under investigation, along with the abbreviations used in the text.

Succinic acid (SA), glutaric acid (GA), and adipic acid (AA) were sourced from Sigma-Aldrich (Darmstadt, Germany). *p*-Toluenesulfonic acid monohydrate was sourced from VWR International GmbH (Darmstadt, Germany). Trimethylolpropane (T, 98%) and pentaerythrirol (P, 98+%) were sourced from Alfa Aesar (Kandel, Germany). All materials were used without further purification, but crushed in a mortar and dried in a vacuum oven at 40 °C before use. Figure 1 shows the structures of the materials used.

### 2.1. Polyester Preparation for Kinetic Assessment

All six polyester resin systems were prepared following the same procedure using an equimolar ratio of acid to hydroxy groups. For example, 23.9 mmol (2.82 g) of succinic acid was mixed with 15.9 mmol (2.14 g) of trimethylolpropane and 0.26 mmol (0.045 g) of *p*-toluenesulfonic acid monohydrate, equating to 0.5 mol% catalyst relative to the OH groups. The dry mixture was then added to a beaker and mixed thoroughly by hand. The required quantities were then transferred into a 40 mL snap cap vial and placed, without the lid, in an oven at a temperature range of 150 °C to 210 °C for up to 4 h (see Table S1 in Supplementary Materials). For each data point, an individual vial containing approximately 5 g of mixture was prepared.

### 2.2. Test Specimen Preparation

**Figure 2:**
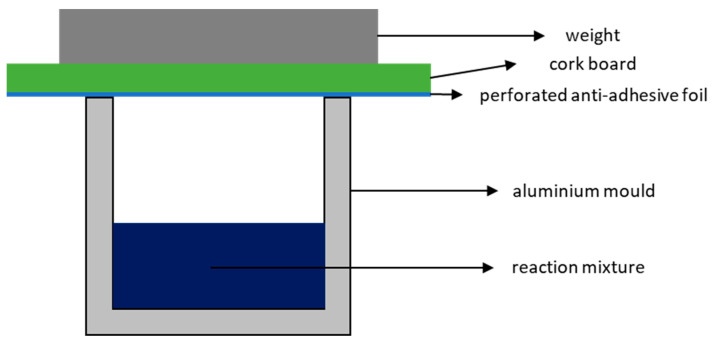
Setup for the production of test specimens in an aluminium mould.

To prepare the test specimens, the desired amount of reaction mixture was weighed using the stoichiometry given above and transferred to preheated, anti-adhesive foil-covered aluminium moulds. The moulds were then covered with a perforated anti-adhesive foil and a cork board to allow the water vapour formed during the reaction to evaporate and then transferred to an oven preheated to the desired reaction temperature (Table S1 in Supplementary Materials). The moulds were then loaded with a weight to prevent the foaming reaction mixture from squeezing out the forms and to ensure a defined shape of the test specimens (Figure 2). To obtain purely white foams and prevent degradation, the moulds were removed from the hot oven after 1 h for high-temperature samples and up to 4 h for low-temperature samples, followed by transferring them to a preheated oven at 130 °C for 4 h to ensure full polymerisation. This method enabled the production of test specimens with minimal variation, achieving deviations in conversion and yield of less than 1%.

### 2.3. ATR-FTIR Spectroscopy

Infrared spectra were recorded on a Spectrum Two with an ATR attachment (Perkin Elmer LAS, Rodgau, Germany). Twenty-four scans were acquired with a resolution of 4 cm^−1^ in a wavenumber range of 400–4000 cm^−1^.

#### 2.3.1. Thermogravimetric Analysis (TGA)

The thermal stability of the final polyester was measured on a TGA-4000 (Perkin Elmer LAS, Rodgau, Germany, temperature resolution ± 1 °C, weight resolution ± 0.1 µg). Approximately 10 mg of the sample was placed into the crucible and heated from 30 °C to 900 °C at a rate of 10 °C/min under a continuous nitrogen flow of 20 mL/min.

#### 2.3.2. Thermal Conductivity Tests

To investigate the thermal insulation properties, the cubic specimens were placed in an insulating frame and placed on a hotplate at 80 °C. The change in surface temperature over time was recorded using a thermal imaging camera FLIR E690 (FLIR Deutschland, Frankfurt am Main, Germany, temperature resolution of ±2 °C) at 5 min intervals.

#### 2.3.3. Scanning Electron Microscope (SEM)

SEM images were recorded using a Thermo Scientific Environmental Scanning Electron Microscope FEI Quattro S (Thermo Fisher Scientific, Fei Deutschland GmbH, Dreieich, Germany) with a point resolution of 1.3 nm (SE low vacuum) and SE, BSE, EDX, and STEM detectors. Prior to imaging, the samples were sputter-coated with a thin conductive layer of gold and platinum (Au/Pt) to enhance surface conductivity and minimise charging effects during electron beam exposure.

#### 2.3.4. Flammability Tests

Flammability tests were carried out in a Testex UL94 combustion chamber (Textex, Dongguan, China) with a methane gas burner at a pressure of 10 mbar and a gas flow rate of 100 mL/min. A blue flame with a flame height of 20 mm and a distance of 10 mm from the test specimen was used (based on the UL94 standard [28]). The flaming time was 2 times 10 s, and for long exposure times, the flaming time was increased until ignition occurred.

## 3. Results and Discussion

**Figure 3:**
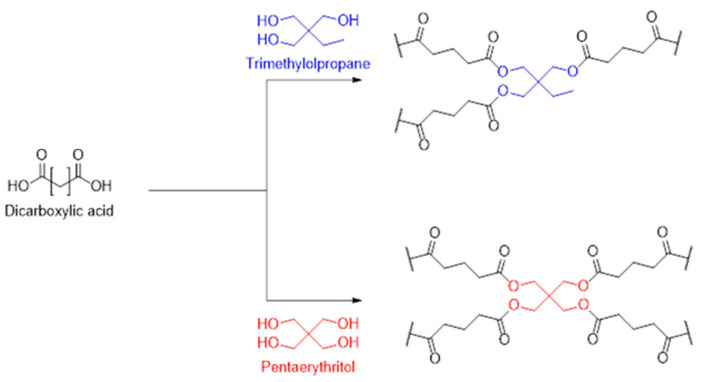
Controlling crosslinking density by using different polyols as crosslinkers in the polycondensation reaction.

The set of acids comprises succinic, glutaric, and adipic acid in order to correlate the influence of chain length with reactivity and the macroscopic appearance of the foams. Trimethylolpropane and pentaerythritol, despite not currently being available from bio-based sources, were selected to check the influence of the crosslinking density without running into selectivity issues, since both contain only primary OH groups (Figure 3). The stoichiometry was always kept at a 1:1 functional group ratio to allow the reaction to potentially reach 100% conversion. This way, differences in the macroscopic properties can more reliably be related to the molecular properties of the components. It was deliberately decided to not use glycerol as a starting material, although it is produced in large quantities in biodiesel production. The reason is that it has two primary and one secondary hydroxy group, which are known to have different reactivities in polycondensation reactions, and this would have made the evaluation and analysis of the results unnecessarily complicated [29].

**Figure 4:**
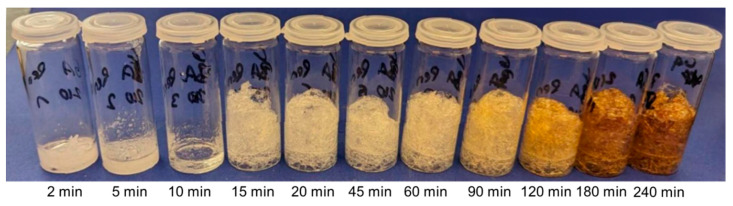
Reaction progress of the glutaric acid–pentaerythritol system at 210 °C (GA-P210).

The esterification releases water, which causes crosslinkable mixtures to foam at high temperatures [30]. The qualitative progress of such a reaction is shown in Figure 4 using the glutaric acid–pentaerythritol system at 210 °C (GA-P210) as an example. Three distinct phases can be identified: Since all compounds are solid at room temperature, the initial phase consists of melting and the formation of a homogeneous liquid. It is important to note that although the compounds are already being converted during melting, as confirmed by IR measurements (vide infra), no foaming occurs in this phase. Following this, the reaction noticeably accelerates, and foaming occurs rapidly (second phase). After prolonged times, the foam begins to discolour, indicating thermal decomposition (third phase). This simple evaluation makes it possible to determine the appropriate reaction time for each reaction temperature and system in order to limit the decomposition reaction.

### 3.1. FTIR Spectra

**Figure 5:**
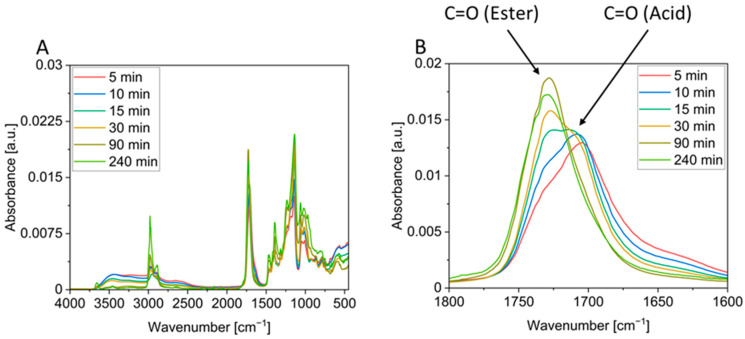
IR spectra of the GA-P210 system and different reaction times (**A**) and the observed signal of the carbonyl band between 1600 cm^−1^ and 1800 cm^−1^ (**B**).

The progress of the reaction was followed using IR spectroscopy (Figure 5), which corroborates the qualitative assessment shown in Figure 4. As all signals changed in intensity during the reaction, the spectra were normalized to the area of all carbonyl bands as these remain the same during the reaction. This reveals a continuous decrease in the acid vibration at 1685 cm^−1^ accompanied by an increase in the ester vibration at 1727 cm^−1^. For example, the ester band clearly increased between 5 and 10 min of reaction time, although foaming had not yet started by 10 min. This confirms the above statement that the compounds are already being converted before foaming begins (cf. Figure 4). Between 10 and 15 min, the ester band becomes clearly visible, which coincides with the onset of foaming. The intensity of this band increases over the next approximately 75 min, although no significant change in the foam appearance can be observed (Figure 4). This corroborates that foaming occurs in a rather limited time frame, and further conversion of the functional groups mainly consolidates the polyester framework surrounding the bubbles. At high temperatures and long reaction times, the intensity of the ester band decreases slightly, which indicates the onset of decomposition. Since the acid band seems to disappear completely, it can be assumed that a high conversion was achieved.

### 3.2. Kinetic Evaluation

**Figure 6:**
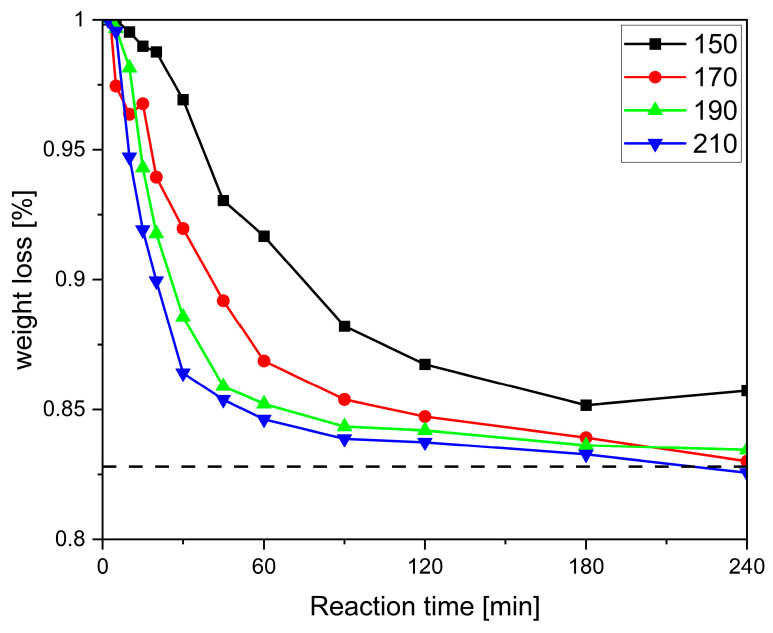
The weight loss as a function of time and temperature recorded from individual experiments in the GA-P system. The dashed line indicates the theoretical mass loss of 82.2 wt%.

To select the proper conditions for the preparation of larger specimens, it is important to quantitatively assess the conversion-time profiles at different temperatures. We chose the interval between 150 and 210 °C in steps of 20 °C. In Fischer esterification, the formation of one molecule of water indicates the conversion of one hydroxy and one acid group to one ester group. Previous studies have monitored the conversion by, e.g., OH-group analysis [26], which in the present case is not feasible as the product becomes increasingly insoluble. Assuming that water is the only volatile compound under the selected reaction conditions, the mass loss over the course of the reaction correlates with the concentration of alcohol, acid, and ester groups, as well as the degree of polymerisation, and can thus conveniently be used to record the conversion-time profiles [31]. Isothermal thermogravimetric analysis seemed to be an ideal method for this, as the reaction can be monitored continuously without interruption. This, however, did not provide the desired reproducible results. The main reason for this was found to be the high-volume stream of nitrogen that flushed the sample chamber in the equipment. Control experiments with pure T at 190 °C revealed that this causes pronounced evaporation even of high-boiling compounds in a short period of time (Figure S1 in Supplementary Materials). As a result, conversion-time curves from individual experiments were recorded gravimetrically, resulting in more consistent trends (Figure 6).

For all systems, a short slow induction period is observed, which becomes shorter with increasing temperature (Figure 6 and Figures S2–S6 in Supplementary Materials). This is again in line with the observations in Figure 4 and is explained by the reactants having to come up to reaction temperature, melt, and mix—or, in the case of pentaerythritol (P), dissolve, as its melting point is above the highest reaction temperature—before the reaction can fully set in. A closer comparison of Figure 4 and Figure 6 reveals that foaming occurs when the conversion rate d*m*/d*t* is at its maximum. It was also observed that for all systems except SA-P (Figure S6 in Supplementary Materials), the mass loss at all four reaction temperatures approached the theoretical value. Thus, the observed degradation, indicated by the increasingly darker colouring of the foams in the vial test (Figure 4) and the decrease in the ester vibration (Figure 5B), does not result in a significantly increased mass loss beyond the theoretical value (cf. Figure 6 and Figures S2–S6 in Supplementary Materials). This indicates that even small amounts of decomposition products in the otherwise colourless foam lead to discolouration. In addition, systems containing pentaerythritol (P) appear to show a faster tendency for degradation at prolonged reaction times, which might be due to the faster reaction and might indicate that degradation is a side reaction of the product.

The reason why the reactions of the SA-P system at lower temperatures do not reach the theoretical mass loss might be that neither of the two compounds melts below 190 °C. Nonetheless, it is interesting to note that the reaction also takes place at these temperatures and a foam is produced albeit with a greater delay. Here, the low molecular weight oligomers act as solvent, facilitating the reaction of the mostly solid reactants. For reaction temperatures above 190 °C, a mass loss higher than calculated was observed, possibly due to the earlier onset of the decomposition of oligomers or unreacted educts.

For the GA-T system (Figure S4 in Supplementary Materials), the conversion-time curves at 150 °C and 170 °C deviate strongly from the expected course. First, a slow melting process is observed, leading to a shift in the reaction towards longer reaction times. After all the reactants have melted, a plateau appears to be reached after approximately 60 min of reaction time. This can be explained by the formation of a polymer layer on the surface, which acts as a lid and hampers the evaporation of water. The layer is not visible in the vial tests (Figure S7 in Supplementary Materials), but can be detected by probing the mixture, e.g., at 90 min with a needle, which reveals a leathery surface with a viscous liquid beneath. As the evaporation of water drives the equilibrium to the product side, a layer with reduced permeability slows down the reaction. With longer reaction times and increasing pressure of the formed water, the layer can be broken up, water vapour is released, and the reaction continues (Figures S3 and S7 in Supplementary Materials).

As stated above, the mass loss determined gravimetrically can be converted to the concentration of the functional groups with the help of the density. However, assessing the density required making some simplifying assumptions. For example, the density could only be determined for those systems that form liquid prepolymers before the foaming process starts, which is not the case for SA-P and AA-P. It was then assumed that the density was approximately constant during the reaction and at the different reaction temperatures. This is, of course, not the case, but it was assumed that the errors were similar for all the systems under investigation and smaller than the uncertainty of the fit, so that the values were comparable within the set under investigation. On paper, the Fischer esterification is first-order in both alcohol and acid, which adds up to a second-order reaction. However, trying to fit a second-order rate law to the data points provides only a poor correlation, even when excluding the values of the initial phase. A first-order rate law, on the other hand, results in an excellent match in most cases and is, consequently, used for the quantitative evaluation of the reaction. The deviation from the classic textbook knowledge is explained as follows:

During polycondensation, a hydroxy group (**A**) reacts with an acid group (**B**) to form an ester group (**E**) and a water molecule as a by-product. However, the progress of the reaction is not monitored as usual via the decrease in reactants, but via the evaporating water. Provided that the protonation of the ester and the proton transfer from **I_1_** to **I_2_** are fast, the formation of water is controlled by two steps: the bimolecular addition of alcohol to the protonated ester (*k*_1_) and the unimolecular elimination of water from **I_2_** (*k*_2_). Both are equilibrium reactions, but it is evident that **I_2_** ⇌ **E** + H_2_O can be shifted to the **I_2_** side by hampering the evaporation of water, as is the case in a highly viscous medium. The elimination then becomes rate-determining, and the overall formation of water appears as unimolecular reaction.

**Figure 7:**
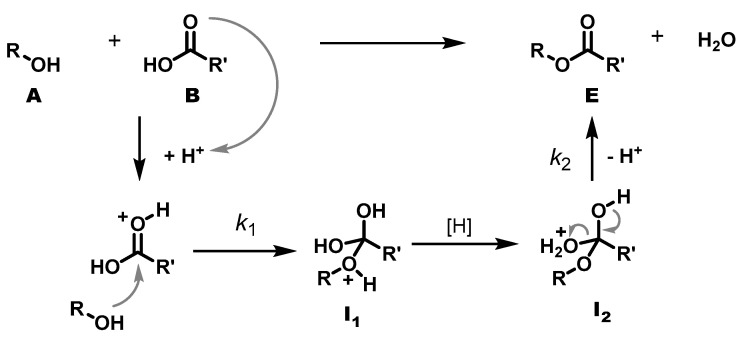
Detailed reaction mechanism of the esterification of an acid (**A**) with a hydroxy group (**B**) to the ester (**E**) via the intermediate states (**I_1_** and **I_2_**).

**Figure 8:**
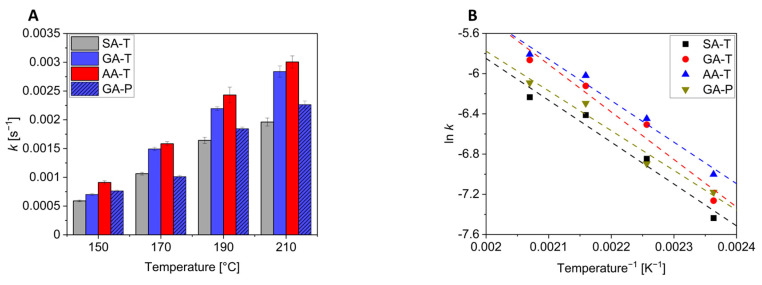
First-order rate constants *k* for all systems under consideration except SA-P and AA-P, which did not form liquid prepolymers (**A**) and the corresponding correlation of ln *k* vs. 1/*T* (**B**). The dashed lines in Figure 8B show the trend lines.

Under the above assumptions and conditions that the elimination of I_2_ to E + H_2_O is the rate determining step leading to a first-order reaction, the polycondensation reactions were analysed using the first-order rate law. Figure 7 shows the apparent rate constants and their dependence on temperature. A table with the values can be found in Supplementary Materials (Table S2). As already seen in Figure 6, the reactions become faster with increasing temperature for all reactions. It is, however, interesting to note that at a given reaction temperature, the values increase in the order AA > GA > SA, i.e., the reaction is faster with higher dicarboxylic acids (Figure 8A). This is due to the increased mobility of the chains as their length increases, which accelerates the reaction by favouring the spatial proximity of the reactive groups. As outlined above, only the GA-P system of the pentaerythritol-containing mixtures could be investigated. Comparing this to the GA-T system reveals another interesting aspect of these mixtures. While at 150 °C the rates of these two systems are comparable, the values for the GA-T system increase faster with increasing temperature than for the GA-P system. This could be due to two effects: First, a denser network is formed with P due to the tetravalent nature, which makes it more difficult for the system to react due to steric hindrance. The second effect might be due to the lower melting point of T compared to P. This results in a more homogeneous and faster mixture, as no dissolution process is included. This can lead to a faster reaction.

Plotting ln *k* vs. 1/T gives straight lines with a coefficient of determination of >0.97 (Figure 8B). The slopes are on the order of −4000 (≈480 kJ/mol) with the exception of the GA-T mixture, which calculates to approximately −4700 (≈560 kJ/mol). These values are, of course, far too high for the activation energy of esterification reactions and may be due to the use of a first-order rate law, the melt reaction, and/or the fact that the reaction was monitored by the evaporating water. As a result, the thermodynamics of the reaction are not discussed further.

Alternatively, a modified temperature correction factor *Q*_20_ was determined according to Equation (1), which indicates an increase in the rate for a temperature jump by 20 K. According to the rule of thumb, an increase in temperature by 10 K should double the rate, so *Q*_20_ should be close to 4. This is not the case for the present reactions, and this might again be due to the reasons given above during the discussion of the thermodynamic parameters. However, the activation energies derived from *Q*_20_ are much more realistic (Table 1) than those extracted from Figure 8B.

(1) $$Q_{20}=\frac{k(T+20)}{k(T)}$$

### 3.3. Test Specimens

The initial attempts to produce test specimens revealed that moulds with the same volume but different geometries lead to different results under otherwise identical conditions. Shallow moulds do not show any pronounced foam formation, which improves as the base area of the mould decreases and the height increases. From the geometries tested, the cube was selected as the shape with the best foam formation and used in further tests.

**Figure 9:**
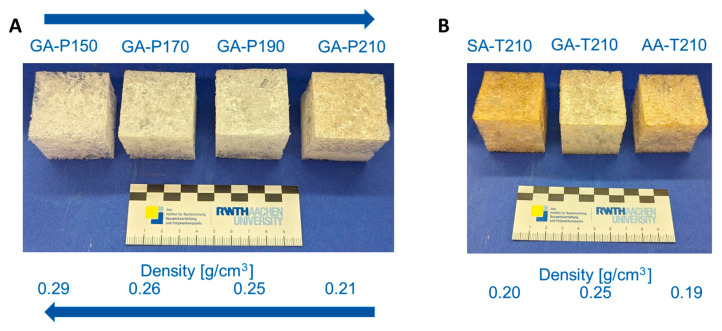
Comparison of the density as a function of the reaction temperature and composition. (**A**): Temperature variation in the GA-P system at 150 °C, 170 °C, 190 °C, and 210 °C. (**B**): Comparison of the chain length of the dicarboxylic acid with trimethylolpropane at 210 °C.

Therefore, to evaluate the physical and thermal properties of the foams, cubic specimens measuring 4 × 4 × 4 cm^3^ were prepared in aluminium moulds lined with an anti-adhesive film (Figure 2). For large-scale preparation, the present set offers several degrees of freedom. Increasing the reaction temperature decreases the density of the foams. For example, in the GA-P system, the density decreases from 0.29 g/cm^3^ at 150 °C to 0.21 g/cm^3^ at 210 °C (Figure 9A). As shown in the reaction kinetics, the reaction rate increases with temperature. This faster reaction leads to the more rapid formation of water, which evaporates and drives the foaming process. Additionally, accelerated curing promotes better stabilization of the resulting foam. Consequently, foam is formed more quickly, achieving a lower density, similar to the effect of an increased blowing agent potential. On the other hand, the variation in the chain length of the acid does not lead to a clear trend in the densities (Figure 9B). The reason why GA-T produces foams with higher densities is unclear, as the only observable difference is that GA is an odd-numbered dicarboxylic acid. However, glutaric acid not being in line with the trend of SA and AA matches the macroscopic appearance of the foams with succinic and adipic acid tending to produce more rigid materials, whereas all foams based on glutaric acid are pliable. A strong influence on the macroscopic appearance is also observed by varying the crosslinking density, as all materials using pentaerythritol (P) showed a significant increase in the apparent hardness. This is explained by the higher crosslinking density compared to T, which results in a more rigid molecular structure and implies that all hydroxy groups of P are active during the polymerisation.

**Figure 10:**
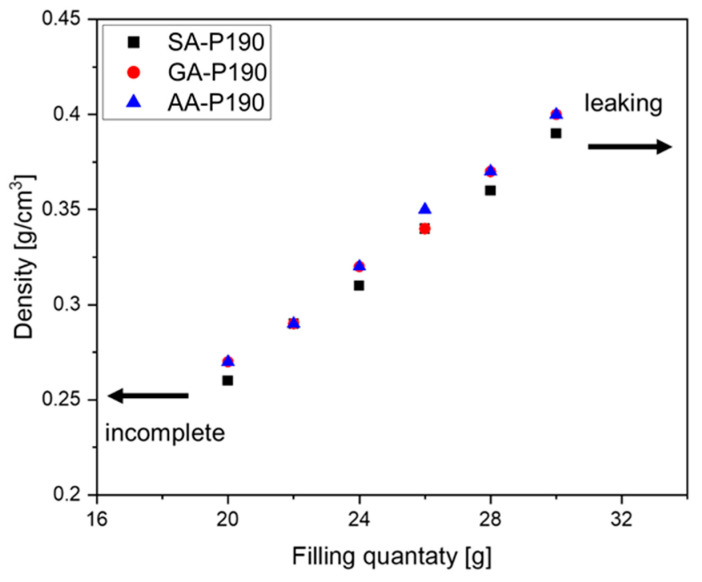
Correlation between the filling quantity and the resulting density of the test specimens of SA-P190, GA-P190, and AA-P190.

Another degree of freedom is the amount of starting material that is put into the moulds at the beginning of the reaction. Figure 10 shows the dependence using the GA-P system at 190 °C as an example. As the foams are produced in a partially closed mould (Figure 2) to allow water vapour to evaporate and the pressure to equilibrate, the resulting density depends on the filling quantity. For our 4 × 4 × 4 cm^3^, the limit lies between incomplete foaming at <20 g and leaking of the material at the top end at >30 g. As a result, foams with densities ranging from 0.26 g/cm^3^ to 0.40 g/cm^3^ could be produced, and each sample matches the theoretical value calculated from the filling mass divided by 64 cm^3^, taking into account the mass loss during polycondensation. The reason why less than 20 g of material does not completely fill the mould with foam is, therefore, that the volume of water vapour available for the foam formation is insufficient, and too much escapes the mould.

**Figure 11:**
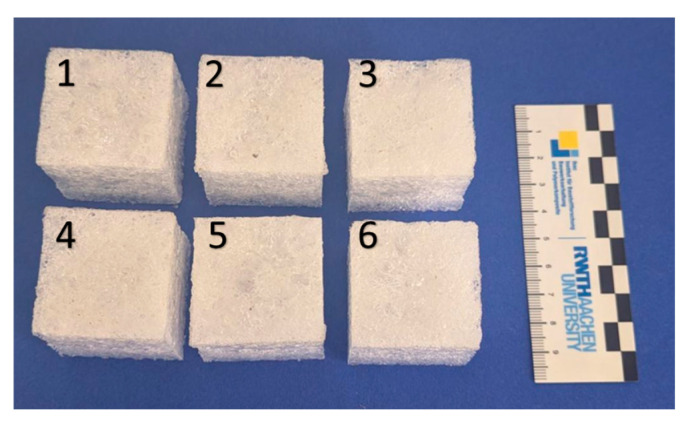
Test specimens produced by varying the filling amounts of 20 g, 22 g, 24 g, 26 g, 28 g, and 30 g (1–6) GA-P190 using the improved foaming-consolidating method.

In order to reduce the discolouration observed with long reaction times and high reaction temperatures, the preparation of the test specimens was slightly modified based on the observations made in Figure 4 and Figure 5. As stated above, foaming occurs in a rather limited time frame, and further conversion of the functional groups mainly consolidates the polyester framework surrounding the bubbles. Thus, when limiting the time at the given reaction temperature to the time interval of foam formation and subsequently consolidating the foam at lower temperatures, e.g., below 130 °C, the discolouration could be completely avoided and the decomposition prevented (Figure 11) compared to the previous test specimen (Figure 9). An overview of all test specimens, including measured mass loss, calculated conversion, and the resulting densities, is provided in Tables S3 and S4 of Supplementary Materials.

### 3.4. Thermogravimetric Examination

Thermogravimetric investigations were carried out in order to investigate the resilience to high temperatures. For this purpose, the thermal decomposition under nitrogen between 30 and 900 °C was investigated. All materials were measured without pre-drying after storage under ambient conditions. It was found that thermal decomposition of the samples of the P-system starts at temperatures of 419 °C (SA-P), 425 °C (GA-P), and 411 °C (AA-P) and is complete at approximately 500 °C, with zero residual mass above 900 °C (Figures S8 and S9 in Supplementary Materials). Complete, i.e., residue-free decomposition is an advantage if biodegradability cannot be fully achieved in a given time frame or if the material is not processed correctly in waste sorting and accidentally ends up in an incineration plant. At temperatures below 200 °C, a greater mass loss was observed for GA-P than for SA-P and AA-P. This could be due to post-polymerisation or an indication of a higher hygroscopicity.

The T-system showed slightly lower decomposition temperatures (370 °C for SA-T, 395 °C for GA-T and 399 °C for AA-T) than the P-system (Figures S10 and S11 in Supplementary Materials). This indicates that the crosslinking density has an influence on thermal stability. The higher crosslinking density of the P-system compared to the T-system results in thermally more stable materials. For the systems containing T, a slight increase in the decomposition temperature to longer chain length was observed, while there was no clear trend for the P-system. Complete degradation was observed for all systems around 500 °C (Table 2). In any case, the decomposition temperatures (onset and complete) of the present materials are considerably higher than those reported for specimens prepared from citric acid and glycerol (onset < 200 °C, complete at approximately 420 °C) [26].

### 3.5. Thermal Conductivity

**Figure 12:**
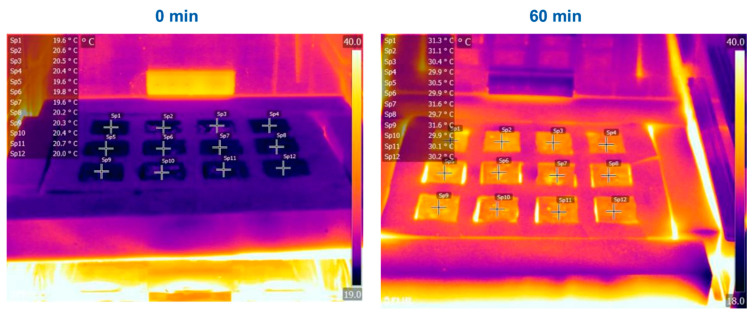
Thermal images of the test specimen set in a PUR-foam frame on a hotplate at 80 °C before (left) and after (right) heating for 60 min. In each row, the reaction temperature increases from 150 °C (left) to 210 °C (right), while from the top to the bottom row, the chain length increases from SA-P (back) to GA-P (middle) to AA-P (front). All samples have a density of 0.25 g/cm^3^.

**Figure 13:**
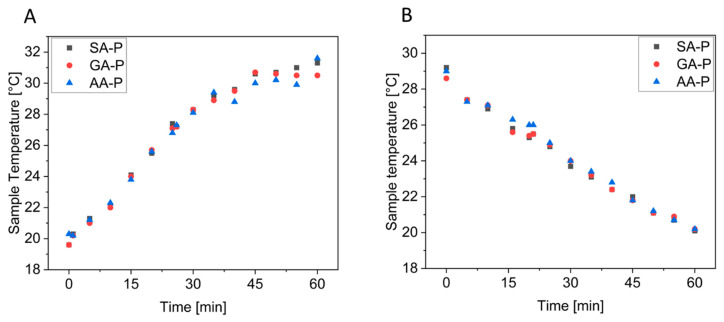
Measured surface temperature of the polyester foams (prepared at 190 °C) during heat treatment of 80 °C (**A**) and cooling at room temperature (**B**), with a density of 0.25 g/cm^3^.

In order to get an indication of the thermal conductivity of the foams, the cubic specimens were placed in a tight-fitting frame made from a commercial polyurethane (PUR) thermal insulation foamboard and placed on a hotplate at 80 °C. The temperature was recorded every 5 min using a thermal imaging camera during heating for 60 min and subsequent cooling (Figures S12 and S13 in Supplementary Materials). The corresponding images at 0 min and after 60 min for the P-system with a density of 0.25 g/cm^3^ are shown in Figure 12, and the course of the sample temperature during heating and cooling is shown in Figure 13. It was, however, not possible with this method to compare the performance of the present foams with a commercial reference, such as EPS or XPS. Thermal imaging reacts very sensitively to changes in the IR reflectivity so that materials with the same temperature but different reflectivity appear to have different temperatures. This effect can clearly be seen in the left image of Figure 12, where the PUR-foam frame appears significantly brighter than the samples, but both are still at room temperature.

It was observed that all materials showed a temperature between 19.6 and 20.7 °C at the beginning (room temperature 20 °C). After heating on a hot plate at 80 °C for 60 min, all materials showed temperatures of up to 29.7 to 31.6 °C. It must be mentioned that the surface of the specimens shows an uneven colouration, which indicates different temperatures. This is mainly due to inhomogeneities due to the open-cell foam structure, which results in a different heat transfer depending on whether there is a cavity or wall structure at the point of measurement. To compensate for this, the point of measurement was kept the same during the experiment. Plotting the measured temperatures over time revealed a linear increase during the first 35 min of heating, which levels off after approximately 50 min (Figure 13). Here, the supply of thermal energy from the hotplate to the sample and its discharge into the surroundings have reached an equilibrium. In contrast, the cooling proceeded linearly until the samples reached room temperature. No difference in thermal conduction or radiation could be observed for the samples in the P-system.

**Figure 14:**
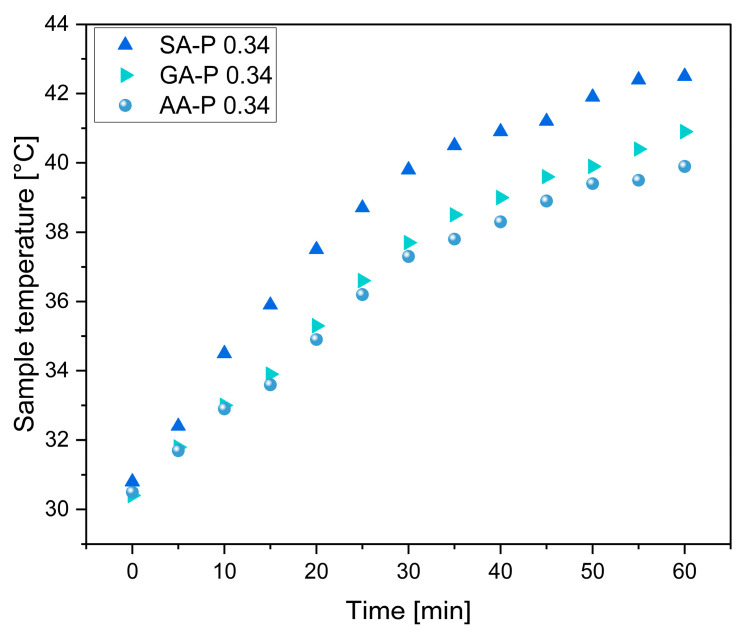
Measured surface temperature of the polyester foams during heat treatment of different test specimens of SA-P, GA-P, and AA-P with a density of 0.34 g/cm^3^.

With the density increasing to 0.34 g/cm^3^, a difference in the measured surface temperature was observed in the various systems (Figure 14, see also Figures S14 to S17 in Supplementary Materials). It was found that the surface temperature of SA-P to GA-P and then to AA-P continued to decrease with increasing chain length. This can be due to better pore stabilisation due to lower steric hindrance in the system with increasing chain length. Hardly any differences were observed between the different densities within a system (Figure S14 in Supplementary Materials). This could be due to different heat reflection, as the differences were measured in different rows to the front and not in one row with the same sample. For further investigation, a different measurement for the thermal conductivity should be considered. A comparison with EPS, XPS, and PUR is shown in Supplementary Materials (Figure S18), where significant heat radiation was also observed.

**Figure 15:**
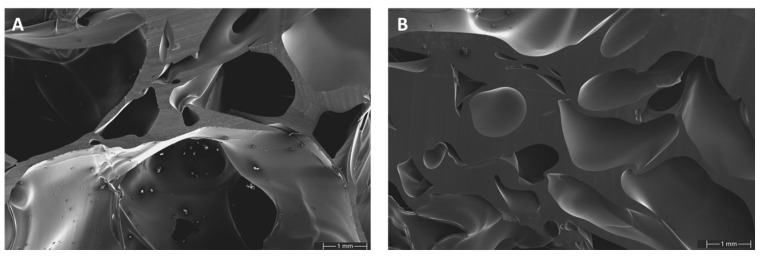
SEM images of the foam structure of GA-P190 with a density of 0.26 g/cm^3^ (**A**) and 0.38 g/cm^3^ (**B**).

SEM images of GA-P190 were taken at two different densities to gain a more detailed understanding of the pore morphology and cell wall structure (Figure 15). At a lower density, the foam exhibits a predominantly open-cell structure with relatively thin cell walls. The majority of the pores appear to be interconnected, although the presence of small, closed cells cannot be entirely ruled out. In contrast, the higher-density sample reveals a more compact microstructure with significantly thicker cell walls and smaller pores. Notably, in both samples, the cell walls display an exceptionally smooth surface and lack any detectable microporosity, indicating a high degree of homogeneity in the polymer phase. These differences indicate that increasing the density of the material limits the extent of foam expansion during polymerisation, leading to reduced pore sizes and improved structural integrity. This correlation between density, pore architecture, and wall thickness plays a key role in determining the mechanical and thermal properties of the resulting foam. Furthermore, at higher magnification, microcrystalline structures become visible. An EDX analysis confirmed the presence of sulphur, suggesting that these microcrystals consist of *p*-toluenesulfonic acid, the catalyst used in the system. Since *p*-toluenesulfonic acid is hydrophilic while the polymer matrix is hydrophobic, the catalyst is pushed out of the matrix during polymerization. Due to the limited mobility caused by the increasing rigidity of the curing polymer network, only small agglomerates can form, resulting in the observed microcrystalline structures (Figure S19 and Table S5 in Supplementary Materials).

### 3.6. Flammability Test

**Figure 16:**
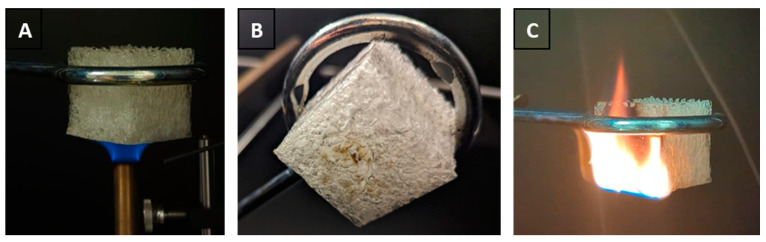
Test specimen during (**A**) and after flaming five times (**B**) for 10 s with a methane flame. Longer exposures of 30 s to the flame ignite the foam (**C**), after which it burns slowly without dripping.

As purely organic materials, foams are bound to be flammable. To investigate whether the foams ignite and to see how they burn, the underside of a test specimen was twice exposed centrally to a methane flame for 10 s (Figure 16A). Although this is a fully organic material with no distinct flame retardant, no ignition was observed, and the foam displayed only a small, slightly charred area where the flame contacted it (Figure 16B). In comparison, EPS and PUR ignited immediately, while XPS ignited after a few seconds of contact with the flame. Burning particles drip off the latter three materials, which poses an enormous risk of spreading fire. In contrast, the polyester foam ignites only after a third, longer exposure of 20 s to the methane flame and then burns only slowly without dripping (Figure 16C). After ignition, the total burning time for one cube is 14 min, while EPS and XPS burn for approximately 10 s and PUR for 20 s. There could be two effects responsible for this superior performance: during foam formation, the expanding water vapour pushes the polymer to the walls, where it reacts to form a dense layer. This smooth but compact crosslinked structure does not melt and provides very few points of attack for the flame. In addition, the pores are filled with water vapour, which acts as a propellant during foam formation and now serves as a flame retardant that impedes ignition and slows down flame propagation.

## 4. Conclusions

When monitoring esterification reactions in the melt by following the evaporation over time, the reaction appears to be first-order. The reason for this is that water elimination in the final mechanistic step is an equilibrium reaction, which is disturbed by the viscous melt. The combination of this quantitative analysis with visual observation of the process revealed that foaming occurs only in a limited time interval around the maximum conversion rate. This means that a certain amount of water must be released very quickly in order to inflate the mixture. Most of the reaction seems to proceed too slowly, causing the polyester framework to solidify, while the water is lost by diffusion without increasing the volume. As expected, molecular parameters such as chain length and crosslinking density influence the macroscopic properties of the foam, although foams based on glutaric acid appear more pliable than those based on succinic or adipic acid. The reason could be the odd number of carbon atoms, which also causes glutaric acid to have a lower melting point than its two homologues. Considering all the observed influences, it is possible to adjust the foam properties depending on the application. These include soft to hard foams with densities ranging from 0.26 to 0.4 g/cm^3^, as well as the control of reaction conditions to prevent thermal decomposition during curing. Thermogravimetric analysis revealed that the foams exhibit increased thermal stability [26] up to approximately 400 °C, with complete decomposition occurring around 500 °C without any residual mass. In addition, the foams are remarkably flame-resistant and withstand multiple brief exposures to a methane flame. They only start to burn after prolonged exposure and then burn approximately 40 times slower than polyurethane foams and approximately 80 times slower than expanded polystyrene. The modular system of bio-based foamed polyesters presents a versatile and sustainable alternative to traditional petrochemical-based foams. As the material properties can be customised through the targeted selection of reactants and the control of reaction conditions, these foams have the potential to meet the growing demand for environmentally friendly materials in various industries.

## Figures and Tables

**Table 1:** *Q*_20_ values according to Equation (1) for the four systems under kinetic evaluation.

System	*T* Interval/°C	*Q*_20_	*EA*/kJ/mol
SA-T	150→170	1.80	45.8
	170→190	1.54	36.8
	190→210	1.19	16.2
GA-T	150→170	2.13	58.9
	170→190	1.47	32.8
	190→210	1.29	23.7
AA-T	150→170	1.74	43.1
	170→190	1.53	36.3
	190→210	1.24	20.0
GA-P	150→170	1.33	22.2
	170→190	1.82	51.1
	190→210	1.23	19.2

**Table 2:** Thermal degradation temperatures of the polyester systems.

Acid	Alcohol	*T*_Onset_ [°C]	*T*_Complete_ [°C]	Mass at 900 °C [wt%]
SA	T	370	492	<0.1
	P	419	506	<0.1
GA	T	395	507	<0.1
	P	425	515	<0.1
AA	T	399	499	<0.1
	P	411	499	<0.1

## Data Availability

Data are available upon reasonable request.

## Supplementary Materials

The following supporting information can be downloaded at https://www.mdpi.com/article/10.3390/polym17091266/s1: Table S1: Overview of the polyester resin matrix., Figure S1: Isothermal TGA of trimethylolpropane (T) at 190 °C showing the mass loss over time due to evaporation in the high-volume nitrogen stream flushing the sample chamber in the TGA equipment. Note that the time to complete evaporation is significantly shorter than the reaction times, Figure S2: SA-T with theoretical mass loss to 82.8 wt%, Figure S3: GA-T with theoretical mass loss to 83.9 wt%, Figure S4: AA-T with theoretical mass loss to 84.8 wt%, Figure S5: SA-P with theoretical mass loss to 80.8 wt%, Figure S6: AA-P with theoretical mass loss to 83.3 wt%, Figure S7. Qualitative impression of the reaction progress for the system glutaric acid-trimethylolpropane at 150 °C (GA-T150), Table S2. First oder rate constants *k* for all systems under consideration except SA-P and AA-P and values of the linear correlation of ln *k* vs 1/*T*, Figure S8: Thermogravimetric examination of SA-P, GA-P and AA-P from 30 to 900 °C, Figure S9: Deviation of the thermogravimetric examination of SA-P, GA-P and AA-P from 30 to 900 °C, Figure S10: Thermogravimetric examination of SA-T, GA-T and AA-T from 30 to 900 °C, Figure S11: Deviation of the thermogravimetric examination of SA-T, GA-T and AA-T from 30 to 900 °C, Table S3: Conversion and mass loss of the prepared samples for kinetic evaluation, Table S4: Resulting densities of the systems SA-P190, GA-P190 and AA-P190, Figure S12: Schematic setup of the analysis of the thermal conductivity, Figure S13: Schematic setup (top) and real setup of the thermal conductivity test using a thermal imaging camera, Figure S14: Measured surface temperature of the polyester foams with different densities of the system SA-P, Figure S15: Measured surface temperature of the polyester foams with a density of 0.29 g/cm^3^, Figure S16: Measured surface temperature of the polyester foams with a density of 0.31 g/cm^3^, Figure S17: Measured surface temperature of the polyester foams with a density of 0.36 g/cm^3^, Figure S18: Infrared images of EPS (top left), XPS (top right), PUR (bottom left), foamed polyester (bottom right) before and after 60 min on a hot plate at 50 °C, Figure S19: SEM image of GA-P190 showing crystalline structures distributed across the surface of the polymer matrix, Table S5: Energy-dispersive X-ray (EDX) analysis of the microcrystalline structures confirmed the presence of sulfur within the material.
