# Supplementary material for: Preparation, Reaction Kinetics, and Properties of Polyester Foams Using Water Produced by the Reaction as a Foaming Agent
Source: Polymers (Basel). 2025 May 6;17(9):1266. doi: 10.3390/polym17091266 (PMC12074305; doi:10.3390/polym17091266)
Supplement: Supplementary file 1 [file polymers-17-01266-s001.zip › polymers-3584799-supplementary.pdf]

Supporting Information

Preparation, reaction kinetics and properties of polyester foams using water produced by the reaction as a foaming agent.

Fabian Weitenhagen and Oliver Weichold*

Institute for Building Materials Research, RWTH Aachen University, Schinkelstraße 3, 52062 Aachen, Germany

* Author to whom correspondence should be addressed: weichold@ibac.rwth-aachen.de

Table S1: Overview of the polyester resin matrix.

	Reaction temperature [°C]			Reaction Time [min]
**Alcohol**	**Succinic acid**	**Glutaric acid**	**Adipic acid**	
Trimethylolpropane	150	150	150	240
	170	170	170	180
	190	190	190	120
	210	210	210	60
Pentaerythrytol	150	150	150	240
	170	170	170	180
	190	190	190	120
	210	210	210	60

Trimethylolpropane shows a weight loss over time at the reaction temperature, making TGA analysis unusable for kinetic studies.

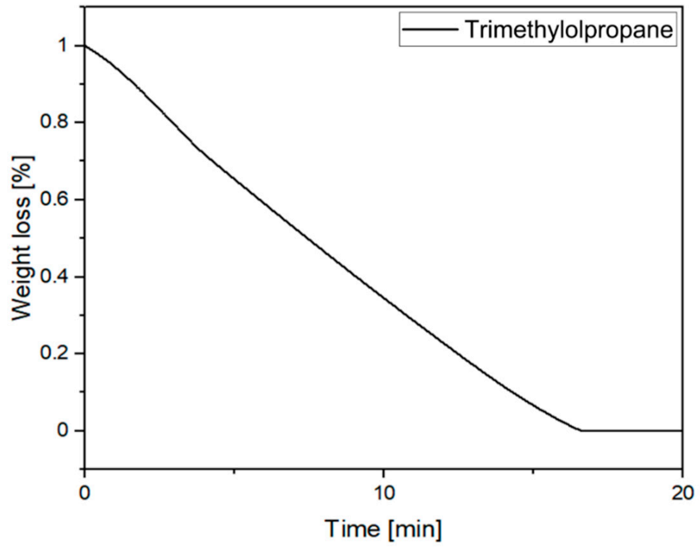

Figure S1: Isothermal TGA of trimethylolpropane (T) at 190 °C showing the mass loss over time due to evaporation in the high-volume nitrogen stream flushing the sample chamber in the TGA equipment. Note that the time to complete evaporation is significantly shorter than the reaction times.

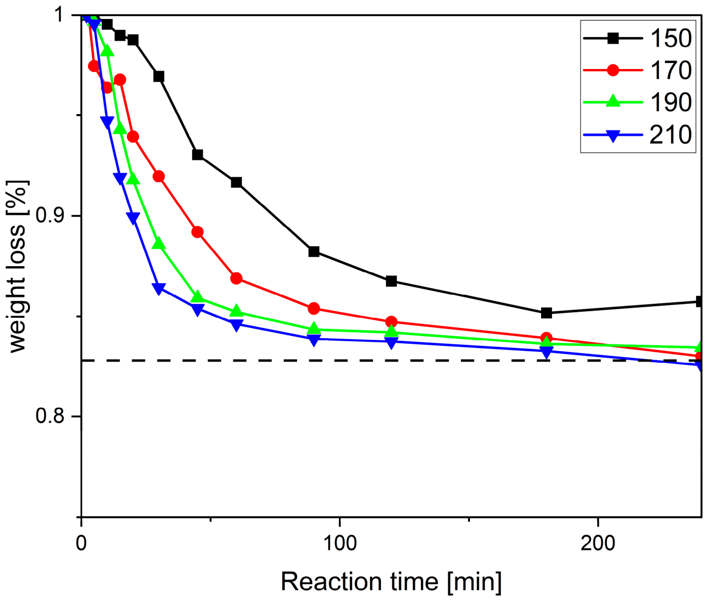

Figure S2: SA-T with theoretical mass loss to 82,8 wt%.

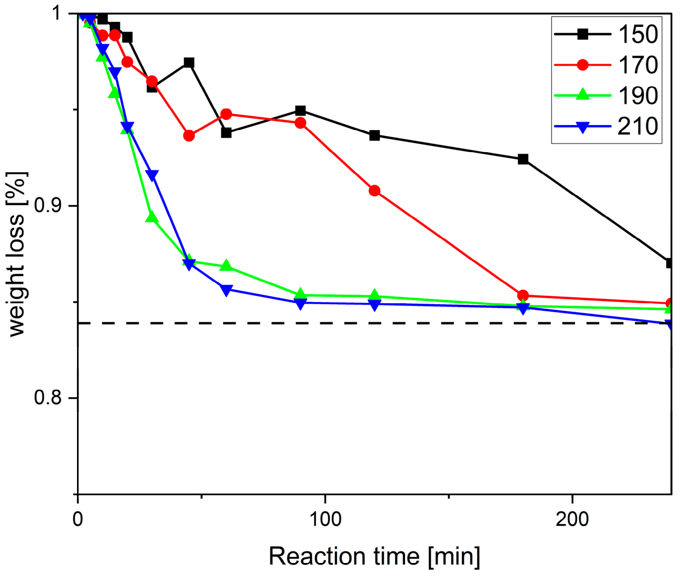

Figure S3: GA-T with theoretical mass loss to 83,9 wt%.

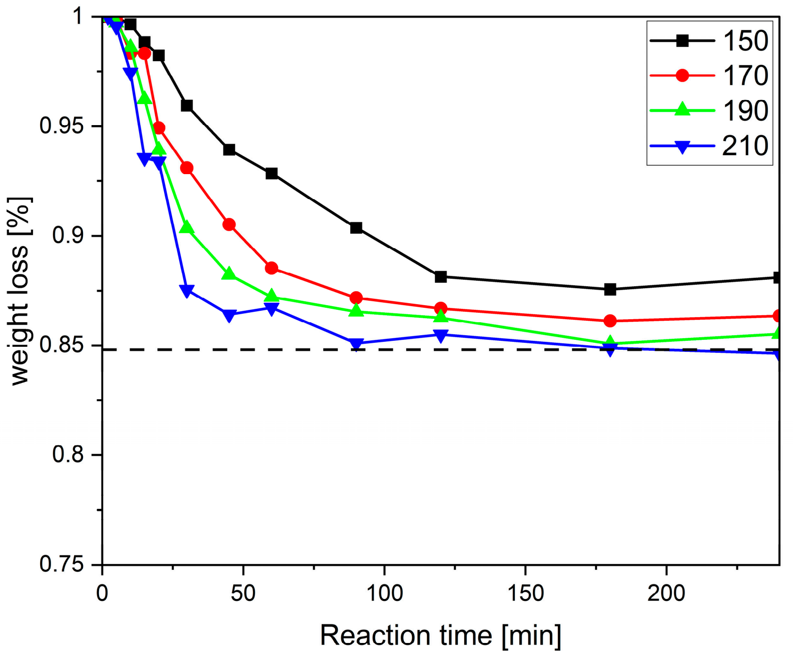

Figure S4: AA-T with theoretical mass loss to 84,8 wt%.

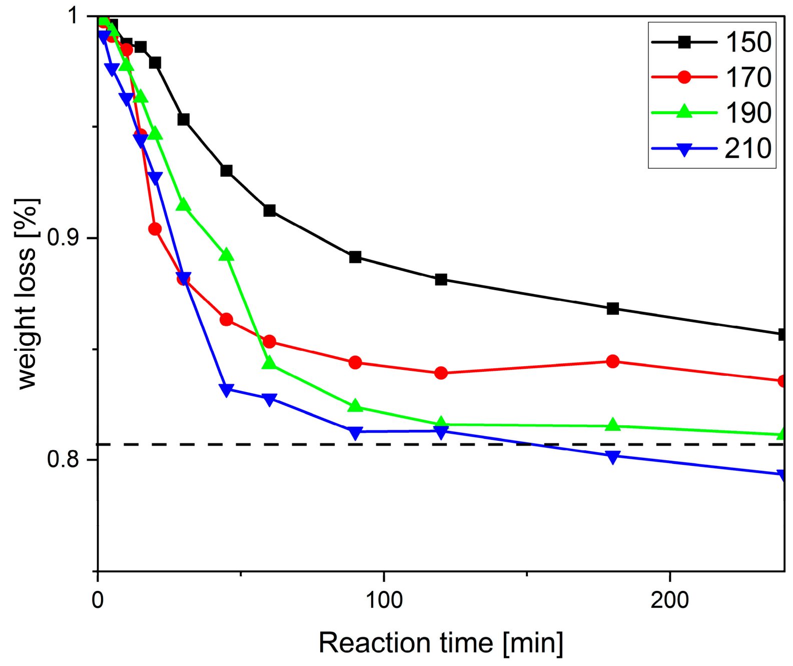

Figure S5: SA-P with theoretical mass loss to 80,8 wt%.

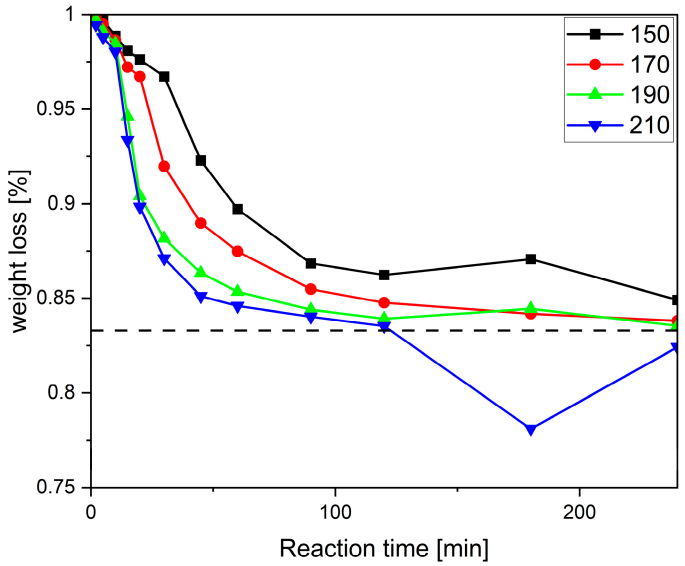

Figure S6: AA-P with theoretical mass loss to 83,3 wt%.

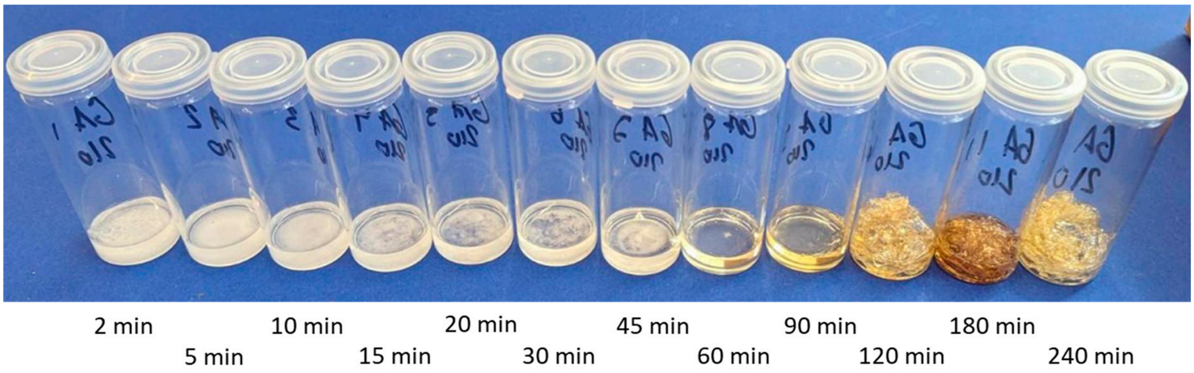

Figure S7. Qualitative impression of the reaction progress for the system glutaric acid-trimethylolpropane at 150 °C (GA-T150).

Table S2. First oder rate constants *k* for all systems under consideration except SA-P and AA-P and values of the linear correlation of ln *k* vs 1/*T*.

System	Temperature [°C]	*k* [s]	slope	Y-axis intercept
SA-T	150	$5,90 \cdot 10^{-4} \pm 1,47 \cdot 10^{-5}$	-4161,6	2,47
	170	$1,06 \cdot 10^{-3} \pm 2,39 \cdot 10^{-5}$		
	190	$1,64 \cdot 10^{-3} \pm 5,37 \cdot 10^{-5}$		
	210	$1,96 \cdot 10^{-3} \pm 6,95 \cdot 10^{-5}$		
GA-T	150	$7,01 \cdot 10^{-4} \pm 1,75 \cdot 10^{-5}$	-4723,1	4,01
	170	$1,49 \cdot 10^{-3} \pm 3,01 \cdot 10^{-5}$		
	190	$2,19 \cdot 10^{-3} \pm 3,28 \cdot 10^{-5}$		
	210	$2,84 \cdot 10^{-3} \pm 9,82 \cdot 10^{-5}$		
AA-T	150	$9,10 \cdot 10^{-4} \pm 2,80 \cdot 10^{-5}$	-4126,2	2,81
	170	$1,58 \cdot 10^{-3} \pm 3,62 \cdot 10^{-5}$		
	190	$2,43 \cdot 10^{-3} \pm 1,38 \cdot 10^{-4}$		
	210	$3,01 \cdot 10^{-3} \pm 1,07 \cdot 10^{-4}$		
GA-P	150	$7,63 \cdot 10^{-4} \pm 1,42 \cdot 10^{-5}$	-3951,7	2,13
	170	$1,01 \cdot 10^{-3} \pm 2,23 \cdot 10^{-5}$		
	190	$1,85 \cdot 10^{-3} \pm 2,90 \cdot 10^{-5}$		
	210	$2,26 \cdot 10^{-3} \pm 6,34 \cdot 10^{-5}$		

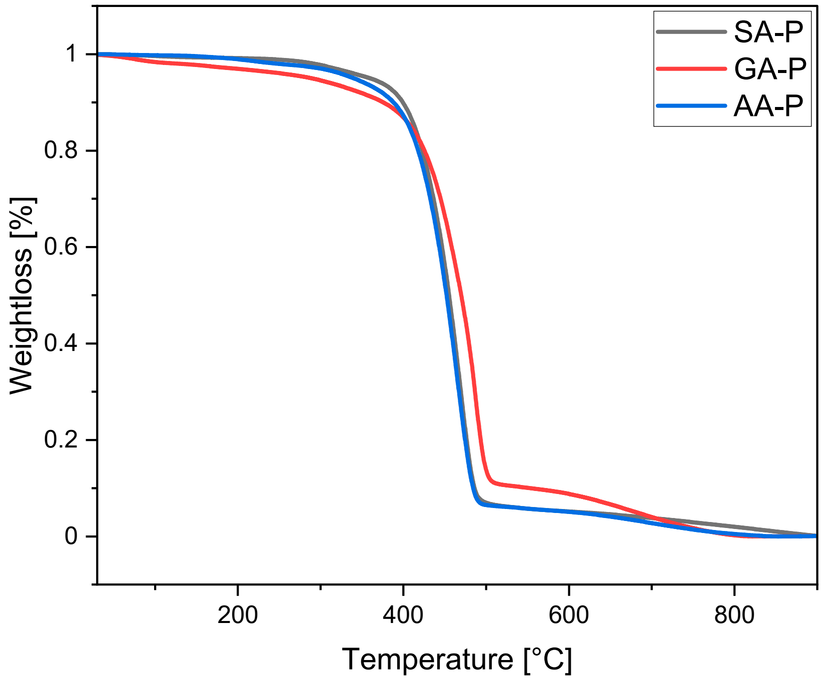

Figure S8: Thermogravimetric examination of SA-P, GA-P and AA-P from 30 to 900 °C.

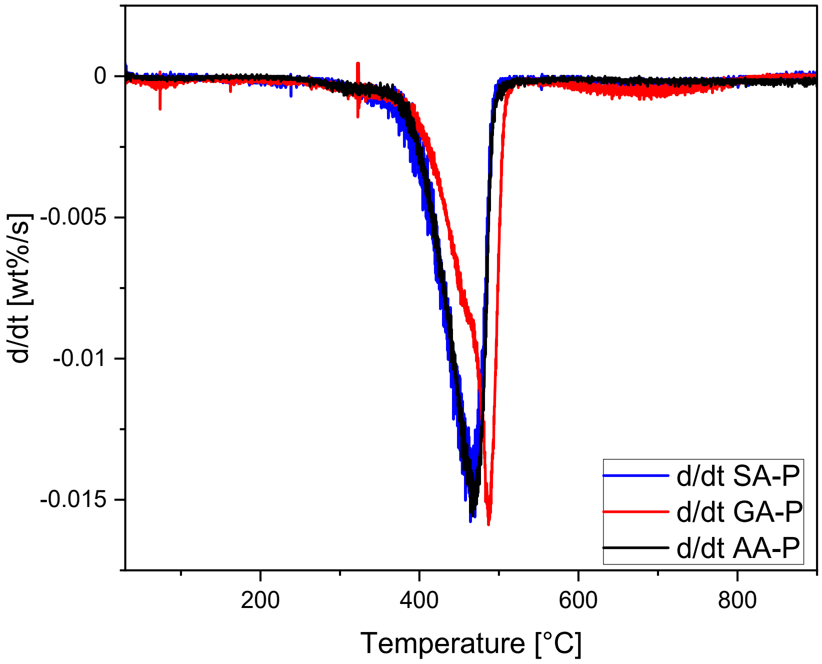

Figure S9: Deviation of the thermogravimetric examination of SA-P, GA-P and AA-P from 30 to 900 °C.

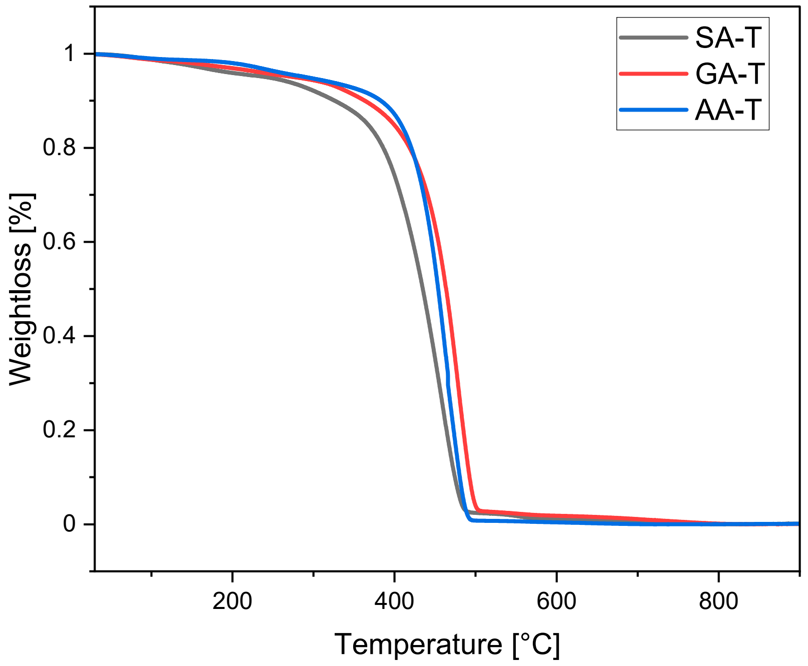

Figure S10: Thermogravimetric examination of SA-T, GA-T and AA-T from 30 to 900 °C.

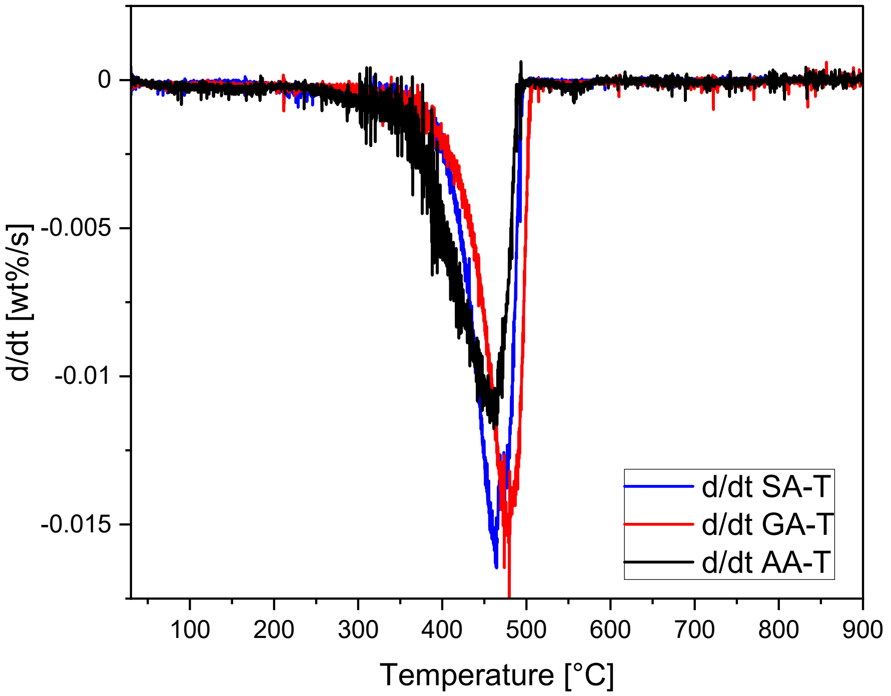

Figure S11: Deviation of the thermogravimetric examination of SA-T, GA-T and AA-T from 30 to 900 °C.

Table S3: Conversion and mass loss of the prepared samples for kinetic evaluation.

System	Reaction temperature [°C]	Mass loss (theo.)	Mass loss (exp.)	Conversion [%]
SA-T	150	17,2	15,0	87,2
	170		16,6	96,5
	190		17,0	98,8
	210		17,4	101
GA-T	150	16,1	13,0	80,7
	170		15,1	93,8
	190		15,4	95,7
	210		16,1	100
AA-T	150	15,2	12,4	81,6
	170		13,9	91,4
	190		14,9	98,0
	210		15,4	101
SA-P	150	19,2	14,3	74,5
	170		16,4	85,4
	190		18,9	98,4
	210		19,7	103
GA-P	150	17,8	16,8	94,4
	170		16,9	94,9
	190		17,3	97,2
	210		18,4	103
AA-P	150	16,7	15,1	90,4
	170		16,2	97,0
	190		16,5	98,8
	210		17,6	105

Table S4: Resulting densities of the Systems SA-P190, GA-P190 and AA-P190.

Weight (before reaction) [g]	$P_{SA\text{-}P190}$ g/cm^3	$\rho_{GA\text{-}P190}$ g/cm^3	$\rho_{AA\text{-}P190}$ g/cm^3
20	0.26	0.26	0.26
22	0.28	0.29	0.29
24	0.31	0.31	0.31
26	0.33	0.34	0.34
28	0.36	0.36	0.37
30	0.39	0.38	0.40

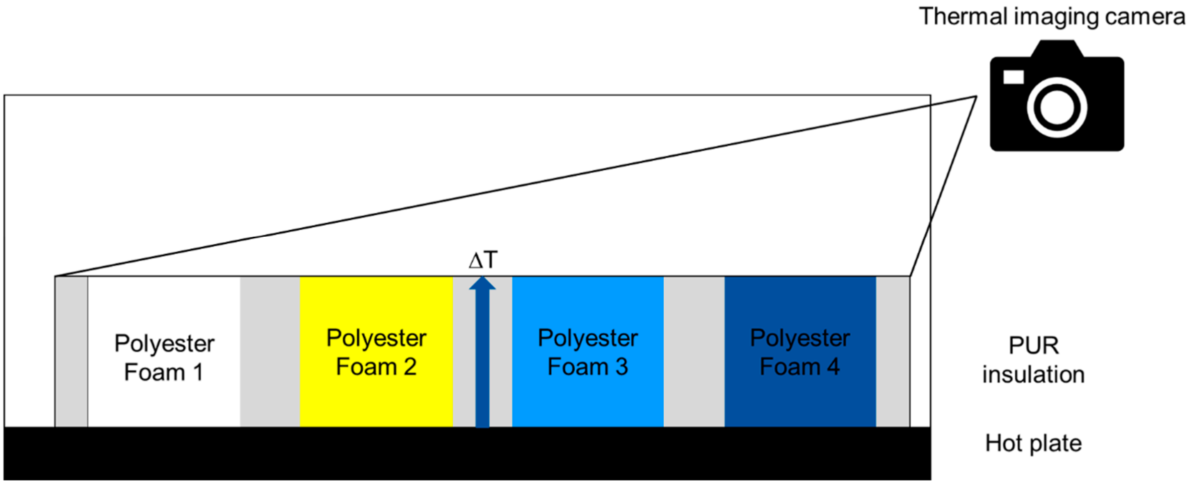

Figure S12: Schematic setup of the analysis of the thermal conductivity.

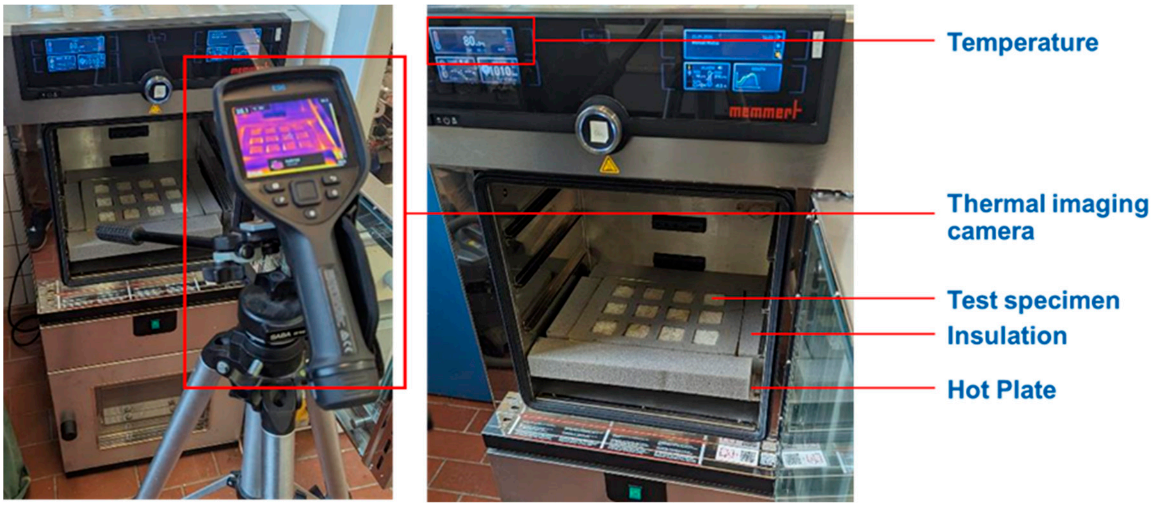

Figure S13: Schematic setup (top) and real setup of the thermal conductivity test using a thermal imaging camera.

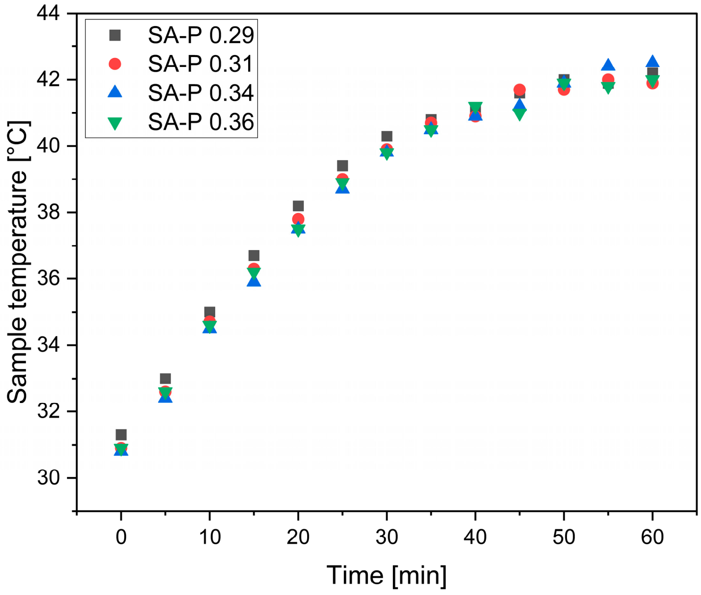

Figure S14: Measured surface temperature of the polyester foams with different densities of the system SA-P.

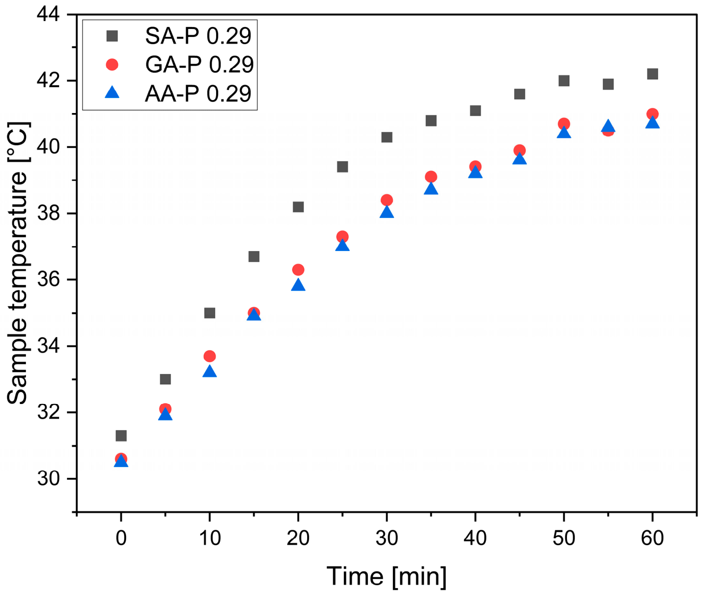

Figure S15: Measured surface temperature of the polyester foams with a density of 0.29 g/cm^3.

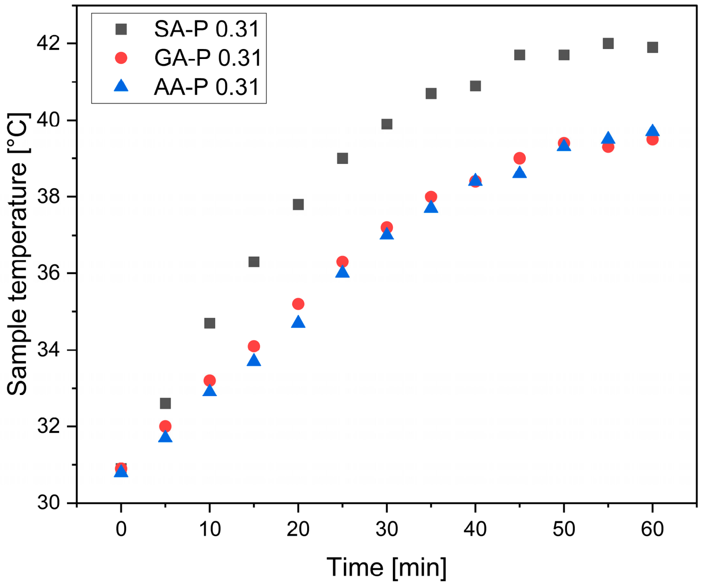

Figure S16: Measured surface temperature of the polyester foams with a density of 0.31 g/cm^3.

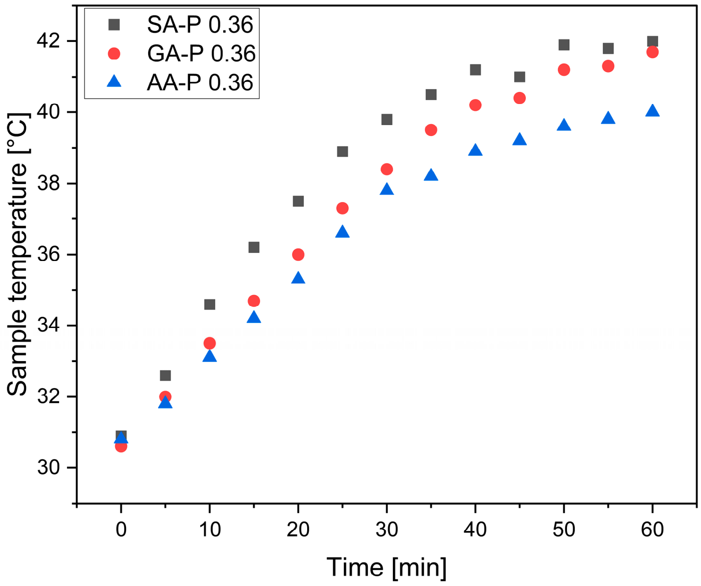

Figure S17: Measured surface temperature of the polyester foams with a density of 0.36 g/cm^3.

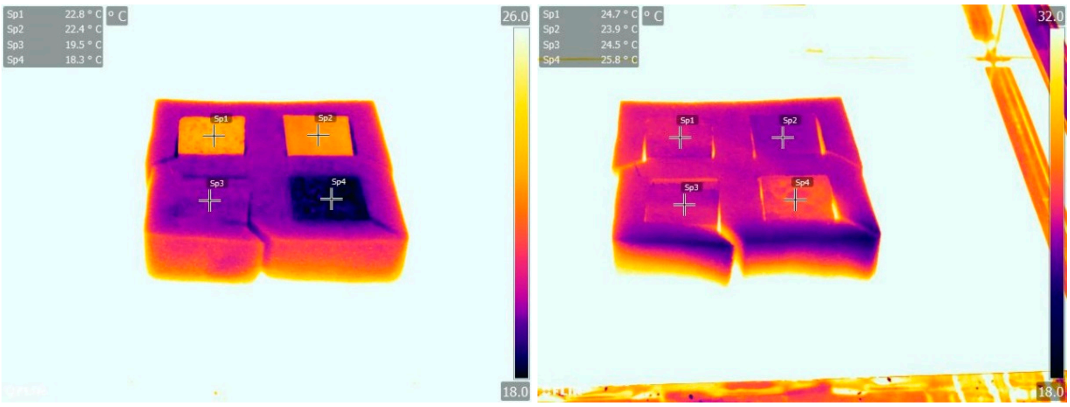

Figure S18: Infrared images of EPS (top left), XPS (top right), PUR (bottom left), foamed polyester (bottom right) before and after 60 min on a hot plate at 50 °C.

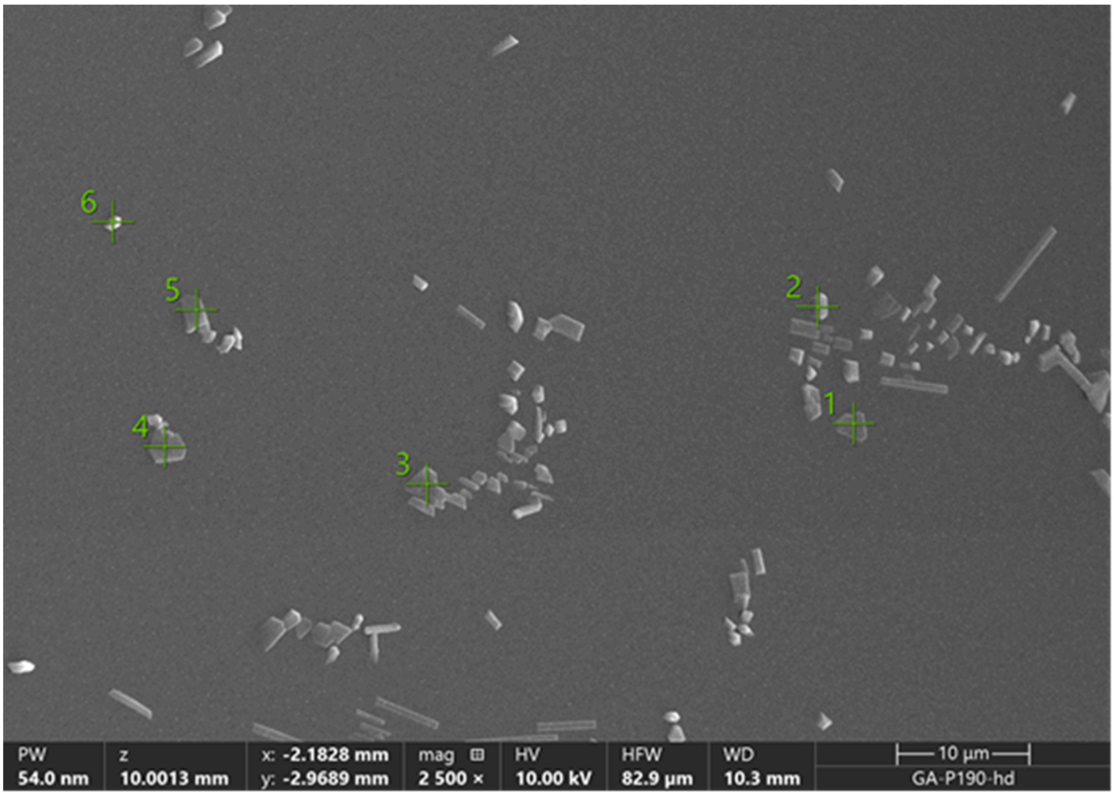

Figure S19: SEM image of GA-P190 showing crystalline structures distributed across the surface of the polymer matrix.

Table S5: Energy-dispersive X-ray (EDX) analysis of the microcrystalline structures confirmed the presence of sulfur within the material.

	Atomic %					
**Element**	**Point 1**	**Point 2**	**Point 3**	**Point 4**	**Point 5**	**Point 6**
C	60.1	58.5	59.0	58.8	57.4	53.7
O	34.8	35.3	35.8	34.3	37.5	37.7
S	2.3	3.7	2.6	4.1	1.9	4.3
Pd	0.9	0.8	0.9	1.1	1.2	1.7
Au	1.9	1.7	1.7	1.7	2.0	2.6
